# Longitudinal association between problematic social media use and school refusal among adolescents: a random intercept cross-lagged panel model

**DOI:** 10.3389/fpsyg.2026.1801922

**Published:** 2026-04-15

**Authors:** Guoqing Min, Jing Yue, Yan Zhang, Juan Yan, Danning Zhang, Kunqiang Yu, Haibin Li

**Affiliations:** 1 Zhejiang Chinese Medical University, Hangzhou, Zhejiang, China; 2 The Second People’s Hospital of Lishui, Wenzhou Medical University, Lishui, Zhejiang, China; 3 Affiliated Mental Health Center & Hangzhou Seventh People’s Hospital, Zhejiang University School of Medicine, Hangzhou, Zhejiang, China; 4 Shandong Mental Health Center, Shandong University, Jinan, Shandong, China

**Keywords:** Chinese adolescents, problematic social media use, RI-CLPM, school refusal, sex differences

## Abstract

**Introduction:**

Problematic social media use (PSMU) and school refusal have become increasingly prevalent during adolescence. However, the longitudinal association between these two constructs remains insufficiently understood, particularly with respect to potential sex differences.

**Methods:**

Using three-wave longitudinal data, this study applied a Random Intercept Cross-Lagged Panel Model (RI-CLPM) to disentangle within-person reciprocal associations between school refusal and PSMU. The sample comprised 1,216 Chinese adolescents (49.5% females; *M* age = 13.60 years, *SD* = 0.76).

**Results:**

RI-CLPM analyses revealed bidirectional within-person effects, such that elevations in school refusal were associated with subsequent increases in PSMU, and vice versa. Multi-group comparisons further demonstrated sex-specific patterns. Specifically, the prospective effect of school refusal on PSMU was more pronounced among females, whereas the predictive effect of PSMU on later school refusal was stronger among males.

**Conclusion:**

These findings underscore the mutually reinforcing nature of school refusal and PSMU during early adolescence and point to the importance of incorporating sex-sensitive components in prevention and intervention efforts.

## 1 Introduction

Problematic social media use (PSMU) refers to excessive social media engagement characterized by addiction-like symptoms that impair daily functioning (Casale, 2020; Marino et al., 2021). Such impairments include academic or occupational difficulties and conflicts with family members and peers (Ren et al., 2026). Core features of PSMU involve poor control over social media use, persistent preoccupation with online activities, and difficulty resisting urges (Casale and Banchi, 2020). Longitudinal evidence indicates that PSMU is an increasing concern among adolescents and young adults. A study of Chinese university freshmen reported that PSMU prevalence increased from 2.5 to 4.7% within 1 year (Zhou et al., 2023). Similarly, a 12-month prevalence rate of 3.5% was reported among Chinese adolescents, with comparable findings in other longitudinal studies (Luo et al., 2021; Lu et al., 2025). Adolescent PSMU has been associated with poor academic performance, sleep problems, depression, and social anxiety (Casale and Fioravanti, 2015; Ahmed et al., 2024). In addition, higher PSMU levels are associated with loneliness, aggressive behaviors, and strained family relationships (Zhou et al., 2023; Lin et al., 2025).

School refusal refers to persistent difficulties attending or remaining in school, often accompanied by avoidance behaviors and emotional distress (Kearney, 2006). It is frequently associated with anxiety, fear, and negative affect (Pérez-Marco et al., 2024). It is highly prevalent among adolescents, with estimates ranging from 28 to 35% in Western and global samples (Kearney and Albano, 2007). Similar prevalence rates have been reported in China, ranging from 22.5 to 30% (Xu et al., 2025). Adolescents exhibiting school refusal are more likely to experience emotional and behavioral difficulties (Chen et al., 2024).

School refusal has been increasingly linked to PSMU, although the direction of this association remains unclear. According to escape-avoidance theory, individuals tend to withdraw from environments that elicit persistent stress or negative emotions (Dinsmoor, 1977). Adolescents experiencing academic pressure or social difficulties at school may therefore rely on social media for distraction and emotional relief (Yildirim Demirdöğen et al., 2024). Social media platforms provide immediacy, perceived control, and accessible social interaction, which may reinforce avoidance-oriented coping patterns (Sun et al., 2023; Wegmann et al., 2023). Over time, repeated reliance on social media to regulate school-related distress may increase vulnerability to PSMU. Conversely, PSMU may also contribute to the development of school refusal. From a self-determination perspective, social media use can satisfy needs for autonomy, competence, and belonging (West et al., 2024). When online experiences become more rewarding than school-related activities, adolescents may show reduced academic motivation and engagement (Montag et al., 2024). Excessive social media involvement may further decrease tolerance for effortful learning and amplify contrasts between online gratification and school demands (Leonhardt et al., 2025). Under such conditions, adolescents may develop stronger reluctance toward school attendance.

Empirical evidence provides direct or indirect support for a reciprocal association between problematic digital behaviors and school functioning. Cross-sectional studies indicate that PSMU and problematic Internet use are associated with truancy and school absenteeism across diverse cultural contexts (Fujita et al., 2022; Kosola et al., 2024; Ren et al., 2025a). Review-level evidence further suggests a consistent association, with problematic Internet use identified as a risk factor for school refusal (Di Vincenzo et al., 2024). However, existing syntheses have not specifically examined school refusal in relation to PSMU. Longitudinal research shows that problematic digital behaviors predict declines in academic functioning and increased absenteeism over time (Masood et al., 2020; Chen, 2022; Amez et al., 2023). Recent longitudinal findings also suggest bidirectional associations between PSMU and broader indicators of school adaptation (Li et al., 2025). Nevertheless, no longitudinal studies have directly tested reciprocal relations between school refusal and PSMU, underscoring an important gap in the literature.

Emerging evidence suggests that the association between school refusal and PSMU may vary by sex. Epidemiological studies consistently indicate that female adolescents report a higher prevalence of PSMU than males (Lu et al., 2025). Moreover, males with elevated PSMU tend to show stronger sensitivity to reward-related features of digital activities (Maza et al., 2023). In contrast, females with PSMU more often exhibit affective dysregulation and mood-related symptoms (Shannon et al., 2022). These sex-specific motivational and emotional patterns may contribute to different pathways linking school-related distress and problematic social media use. Sex differences have also been observed in school attendance problems. Female students generally report higher levels of school non-attendance than males (Havik et al., 2015). Latent profile analyses further suggest that females are overrepresented in mixed or multifaceted school refusal profiles, although effect sizes are relatively small (Gonzálvez et al., 2020). This pattern indicates limited overall sex differences but greater complexity in school refusal presentations among females.

Although no study has directly tested sex as a moderator between school refusal and PSMU, existing evidence suggests plausible sex-specific pathways. School-related distress may exert a stronger influence on PSMU among females due to greater emotional vulnerability (Thorisdottir et al., 2020). Conversely, PSMU may more strongly predict school-related difficulties among males, given their higher prevalence and reward sensitivity (Cardoso Melo et al., 2023). Whether these sex-specific associations unfold longitudinally remains unclear. Therefore, the present study examines sex differences in the longitudinal association between school refusal and PSMU to inform sex-sensitive prevention efforts.

To advance understanding of the developmental dynamics between PSMU, the present study employed a three-wave longitudinal design and applied a Random Intercept Cross-Lagged Panel Model (RI-CLPM). Although traditional Cross-Lagged Panel Models (CLPM) have been widely used to examine reciprocal longitudinal associations, they do not distinguish stable between-person differences from within-person fluctuations over time. As a result, cross-lagged effects estimated by CLPM may conflate trait-like individual differences with dynamic within-person processes, potentially leading to biased or inflated estimates of temporal associations. In contrast, the RI-CLPM separates stable between-person variance (i.e., time-invariant individual differences) from within-person deviations around individuals’ own expected levels. Accordingly, the study aimed to clarify the reciprocal within-person temporal associations between school refusal and PSMU. Three hypotheses were proposed: (H1) within-person increases in school refusal would predict subsequent within-person increases in PSMU; (H2) within-person increases in PSMU would predict subsequent within-person increases in school refusal; (H3) the reciprocal within-person associations between school refusal and PSMU were expected to differ by sex. By examining these hypotheses, the study seeks to provide deeper evidence regarding the short-term developmental association between school refusal and PSMU and to inform the development of sex-sensitive prevention and intervention strategies.

## 2 Methods

### 2.1 Participants and data collection

This one-year longitudinal study was conducted in four middle schools located in Zhejiang Province, China. Data were collected at three 6-month intervals: Time 1 (T1; September 2023), Time 2 (T2; March 2024), and Time 3 (T3; September 2024). The target population consisted of adolescents who reported using social media within the past month. Participants were recruited using convenience sampling in collaboration with school administrators.

Data collection was carried out during regular classroom sessions by trained research assistants. Prior to each wave of data collection, students and their parents or legal guardians were provided with detailed information regarding the study’s purpose, procedures, voluntary nature, and confidentiality protections. Written informed consent was obtained from parents or legal guardians, and written assent was obtained from all participating students. Students were informed that their participation was voluntary and that non-participation would not affect their academic evaluations or school records.

To enable longitudinal matching across waves, participants reported their school identification numbers. These identifiers were encrypted for matching purposes and removed from the analytical dataset after matching to ensure anonymity. An information sheet describing participants’ rights and data protection procedures was distributed before survey administration. Ethical approval for the study was granted by the institutional ethics committee of The Second People’s Hospital of Lishui of Wenzhou Medical University (approval no. 20241205-01).

At baseline (T1), 1,341 adolescents completed the survey. By the final wave (T3), 1,216 participants remained in the study (49.5% females; *M*age = 13.60 years, *SD* = 0.76).

### 2.2 Measures

#### 2.2.1 Background factors

Background factors were collected, including age, sex, self-reported academic performance, perceived family financial level, single-parent family status, and social media use duration.

#### 2.2.2 School refusal

School refusal was assessed using the School Refusal Assessment Scale–Revised (SRAS-R; Kearney, 2002). The instrument includes 24 items designed to capture functional patterns of school refusal behavior. It assesses four dimensions: avoidance of negative affect, escape from aversive situations, attention seeking, and pursuit of tangible rewards. Example items include “How often do you have bad feelings about going to school because you are afraid of something related to school?” (avoidance of negative affect) and “How often do you feel you would rather be with your parents than go to school?” (attention seeking). Participants rated how frequently each situation applied to them. Responses were recorded on a seven-point scale ranging from 0 (never) to 6 (always). Higher scores indicate greater levels of school refusal behavior. The Chinese version of the SRAS-R has demonstrated acceptable reliability and validity among adolescent samples (Zheng et al., 2026). In the present study, the Cronbach’s *α* was 0.88 at T1, 0.91 at T2, and 0.93 at T3.

#### 2.2.3 PSMU

PSMU was assessed using The Bergen Social Media Addiction Scale (BSMAS; Andreassen et al., 2016). The scale includes six items assessing core addiction-related features of PSMU. These features include salience, mood modification, tolerance, withdrawal, conflict, and relapse. An example item is “How often do you feel an urge to use social media more and more?” Participants rated each item on a five-point scale ranging from 1 (very rarely) to 5 (very often). Total scores were obtained by summing all items, with higher scores reflecting more severe PSMU. The possible score range was from 6 to 30. Previous research has demonstrated satisfactory reliability and validity of the Chinese version among adolescent and university samples (Yam et al., 2019). Following prior studies, a cut-off score of 24 was applied to identify individuals with elevated PSMU (Luo et al., 2021; Casale et al., 2023). In this study, the Cronbach’s α was 0.83 at T1, 0.86 at T2, and 0.88 at T3.

### 2.3 Data analysis

Attrition analyses were conducted to compare adolescents who completed all three waves with those who withdrew during follow-up. Independent-samples *t* tests were used to examine sex differences in school refusal and PSMU. Pearson correlation analyses were performed to explore associations among the main study variables. These preliminary analyses were conducted using SPSS version 28.0, applying listwise deletion for missing data. Because these analyses were descriptive in nature and involved a relatively small proportion of missing values, listwise deletion was considered acceptable at this stage.

To examine the temporal consistency of the measurement models, longitudinal measurement invariance tests were conducted for both school refusal and PSMU. Model fit was evaluated using commonly accepted criteria, including CFI and TLI values of 0.90 or higher, and RMSEA and SRMR values of 0.08 or lower (Kline, 2016; Lu et al., 2024). Metric and scalar invariance were assessed by comparing nested models, with changes of ΔCFI ≤0.01 and ΔRMSEA ≤0.015 indicating acceptable invariance (Ren et al., 2025b). The results supported stable factor structures over time for both constructs.

Reciprocal relations between school refusal and PSMU were examined using RI-CLPM. An initial model was first estimated in which all structural paths were freely estimated. Subsequently, a constrained model was estimated in which the autoregressive and cross-lagged paths were restricted to be equal across time. After identifying the best-fitting model, covariates were added as predictors of the between-person random intercepts, including age, sex, academic performance, perceived family economic status, and single-parent household status. Social media use duration was included as a time-varying within-person covariate. A multi-group RI-CLPM approach was used to test potential sex differences in the longitudinal associations. A freely estimated model was compared with a constrained model in which cross-lagged paths were fixed to equality across sex. Significant chi-square differences were interpreted as evidence of sex-specific effects.

All structural models were estimated using Mplus version 8.3. Full Information Maximum Likelihood (FIML) estimation was applied to handle missing data. FIML was selected for the structural models because it allows the use of all available data under the assumption of missing at random and provides less biased parameter estimates compared to listwise deletion. Statistical significance was evaluated using two-tailed *p* < 0.05.

## 3 Results

### 3.1 Attrition analyses

As shown in Table 1, participants who completed all three waves did not differ significantly from those who discontinued participation on background variables or key study variables.

**Table 1:** Attrition analyses at baseline.

Variables	Follow-up (*n* = 1,216)		Lost to follow-up (*n* = 125)		*p*
Categorical variables	*n*	%	*n*	%	
Sex					0.557
Males	611	50.5	66	53.2	
Females	600	49.5	58	46.8	
Self-reported academic performance					0.223
Bottom 20%	157	13.0	19	15.3	
21st–40th percentile	258	21.4	24	19.4	
41st–60th percentile	311	25.7	27	21.8	
61st–80th percentile	280	23.2	24	19.4	
Top 20%	202	16.7	30	24.2	
Perceived family financial level					0.243
Below average	263	21.6	32	25.6	
Average	764	62.8	69	55.2	
Above average	189	15.5	24	19.2	
Single-parent family status					0.259
Yes	180	14.8	13	10.4	
No	919	75.6	96	76.8	
Choose not report	117	9.6	16	12.8	
Social media use duration					0.793
<4 h	225	18.5	26	20.8	
4–8 h	238	19.6	20	16.0	
8–12 h	326	26.8	28	22.4	
12–16 h	275	22.6	30	24.0	
>16 h	152	12.5	21	16.8	

Continuous variables	Mean	SD	Mean	SD	*p*
Age	13.60	0.76	13.54	0.72	0.379
School refusal (0–144)	49.67	17.30	48.20	17.08	0.364
PSMU (6–30)	11.43	4.38	11.50	5.07	0.869

PSMU = problematic social media use.

### 3.2 Descriptive analyses

Among participants who completed all three waves, the mean baseline age was 13.60 years (SD = 0.76), with females comprising 49.5% of the sample. Approximately one quarter of participants (25.7%) reported academic performance within the 41st–60th percentile range. Most adolescents perceived their family economic status as average (62.8%). A total of 14.8% of participants reported living in single-parent households. About one quarter of the sample (26.8%) reported using social media for 8–12 h per week.

At baseline, the mean scores (SDs) for school refusal (range: 0–144) and PSMU (range: 6–30) were 49.67 (17.30) and 11.43 (4.38), respectively. Such information was also presented in Table 1.

### 3.3 Sex differences in key study variables

Males showed significantly lower mean scores than females in school refusal and PSMU across all three waves (Table 2).

**Table 2:** Sex differences in main variables.

Variable	Males (Mean, SD)	Females (Mean, SD)	*t*	*p*
School refusal at T1	47.60 (17.02)	51.65 (17.17)	−4.12	<0.001
School refusal at T2	46.66 (18.48)	52.03 (18.67)	−5.02	<0.001
School refusal at T3	47.35 (22.32)	54.47 (22.16)	−5.56	<0.001
PSMU at T1	11.09 (4.44)	11.77 (4.44)	−2.78	0.005
PSMU at T2	11.56 (4.54)	12.65 (5.17)	−4.24	<0.001
PSMU at T3	12.10 (5.17)	13.15 (4.84)	−3.83	<0.001

PSMU = problematic social media use.

### 3.4 Longitudinal invariance test

Table 3 presents the longitudinal measurement invariance results for school refusal and PSMU. Configural models demonstrated acceptable fit across waves, indicating stable factor structures. Metric invariance was supported, as changes in fit indices remained within recommended thresholds (ΔCFI ≤ 0.01; ΔRMSEA ≤ 0.015), suggesting consistent factor loadings over time. Scalar invariance was also established (ΔCFI ≤ 0.01; ΔRMSEA ≤ 0.015), indicating equivalence of item intercepts across measurement occasions.

**Table 3:** Longitudinal invariance test.

Variables	Model	CFI	TLI	RMSEA	SRMR	ΔCFI	ΔRMSEA	ΔSRMR
School refusal	Configural invariance	0.945	0.932	0.038	0.044	–	–	–
	Metric invariance	0.943	0.929	0.032	0.045	0.002	0.006	0.001
	Scalar invariance	0.944	0.927	0.030	0.041	0.001	0.002	0.004
PSMU	Configural invariance	0.967	0.959	0.021	0.028	–	–	–
	Metric invariance	0.963	0.957	0.021	0.025	0.004	0.000	0.003
	Scalar invariance	0.960	0.955	0.023	0.028	0.003	0.002	0.003

PSMU = problematic social media use.

### 3.5 Correlations

As demonstrated in Table 4, school refusal showed significant positive correlations with PSMU across the three waves, with correlation coefficients ranging from 0.28 to 0.58 (all *p* < 0.001).

**Table 4:** Correlations.

Variable	1	2	3	4	5	6
School refusal at T1	1					
School refusal at T2	0.52***	1				
School refusal at T3	0.49***	0.62***	1			
PSMU at T1	0.43***	0.31***	0.28***	1		
PSMU at T2	0.36***	0.46***	0.34***	0.58***	1	
PSMU at T3	0.31***	0.40***	0.48***	0.49***	0.57***	1

***p < 0.001. PSMU = problematic social media use.

### 3.6 RI-CLPM

**Figure 1:**
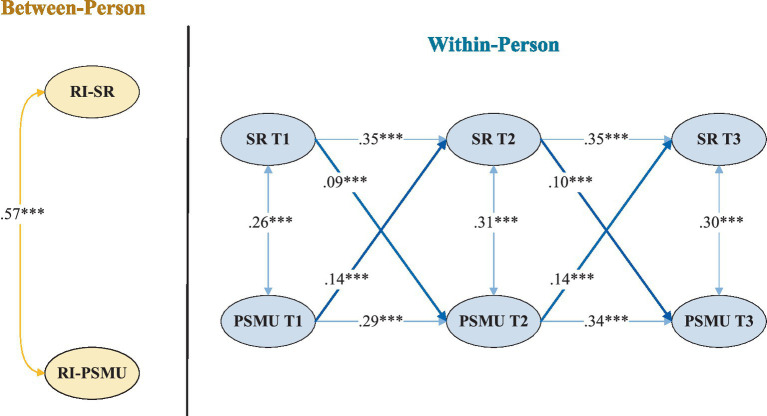
Random intercept cross-lagged panel model for school refusal and PSMU. SR = school refusal; PSMU = problematic social media use. ***p < 0.001.

RI-CLPM demonstrated a good model fit with the data (*χ*^2^(8) = 21.93; CFI = 0.986; TLI = 0.974; RMSEA = 0.021; SRMR = 0.026). As shown in Figure 1, within-person autoregressive paths were significant for both school refusal and PSMU.

Regarding cross-lagged effects, higher levels of school refusal predicted subsequent increases in PSMU across both intervals (T1 → T2: *β* = 0.09, *p* < 0.001; T2 → T3: *β* = 0.10, *p* < 0.001). Conversely, PSMU also prospectively predicted higher levels of school refusal over time (T1–T2 and T2–T3: *β* = 0.14, *p* < 0.001).

Within-person residual correlations between school refusal and PSMU were positive and significant across all time points (*r*: 0.26–0.31). At the between-person level, the random intercepts of school refusal and PSMU were moderately and positively correlated (*r* = 0.57).

### 3.7 Multi-group RI-CLPM

**Figure 2:**
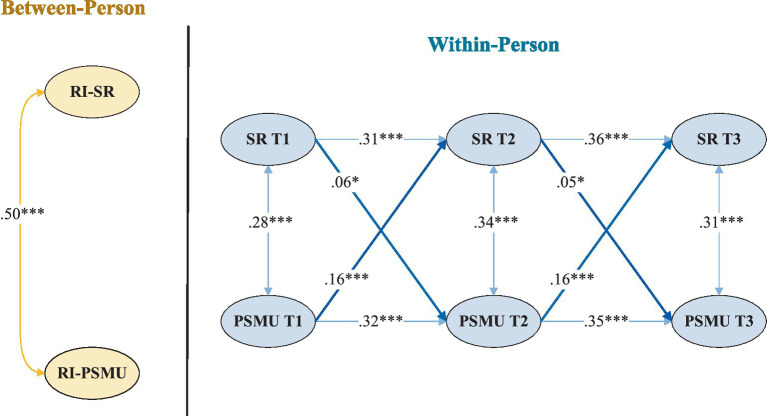
RI-CLPM path diagram for males. SR = school refusal, PSMU = problematic social media use. *p* < 0.01, ***p < 0.001.

**Figure 3:**
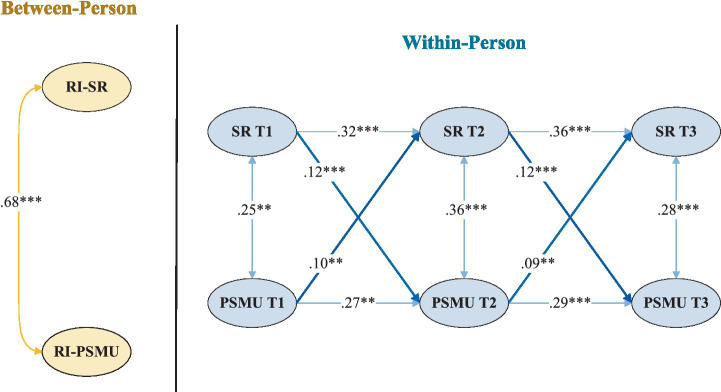
RI-CLPM path diagram for females. SR = school refusal, PSMU = problematic social media use. *p* < 0.01, *p* < 0.001.

A multi-group RI-CLPM was conducted to examine sex differences in the longitudinal associations between school refusal and PSMU. First, an unconstrained model allowing all autoregressive and cross-lagged paths to vary by sex demonstrated good fit to the data (*χ*^2^(12) = 27.44, CFI = 0.976, TLI = 0.962, RMSEA = 0.036, SRMR = 0.029). Next, a constrained model was estimated in which cross-lagged paths were fixed to equality across sexes. This model showed a poorer fit (χ^2^(18) = 68.79, CFI = 0.955, TLI = 0.941, RMSEA = 0.062, SRMR = 0.054). A Chi-square difference test confirmed that constraining the cross-lagged paths significantly reduced model fit (Δχ^2^(6) = 41.35, *p* < 0.001), indicating sex-specific within-person associations (Figures 2, 3).

Further analyses revealed significant sex differences in both cross-lagged pathways. For the path from school refusal to later PSMU, a Wald test indicated stronger effects among females than males (Wald = 7.64, *p* = 0.005). School refusal predicted subsequent PSMU at both intervals for males (*β* = 0.06 and 0.05) and females (*β* = 0.10 and 0.11), with consistently larger coefficients among females. Sex differences were also observed in the reverse direction. PSMU predicted later school refusal for both males and females, but the effects were stronger among males (Wald = 8.42, *p* = 0.004). Specifically, PSMU showed robust predictive effects for males (*β* = 0.16 and 0.13), whereas weaker yet significant effects were observed for females (*β* = 0.10 and 0.09).

## 4 Discussion

The present study employed a three-wave RI-CLPM to examine within-person associations between school refusal and PSMU among Chinese adolescents. By disentangling stable between-person differences, the study clarifies how temporary changes in one behavior predict subsequent changes in the other. The results revealed reciprocal longitudinal relationships. Specifically, when adolescents experienced higher-than-usual school refusal relative to their own typical level, they were more likely to report subsequent increases in PSMU at the next wave. Likewise, when adolescents reported higher-than-usual PSMU, they were more likely to experience subsequent increases in school refusal relative to their own usual level. Multi-group analyses further showed that the strength of these reciprocal pathways differed by sex. The pathway from school refusal to later PSMU was stronger among females, whereas the reverse pathway from PSMU to school refusal was stronger among males. These findings suggest that school refusal and PSMU may form a transactional cycle during adolescence, while also highlighting sex-specific developmental pathways that may require differentiated prevention and intervention approaches.

### 4.1 The reciprocal association between school refusal and PSMU

At the within-person level, school refusal significantly predicted PSMU at the subsequent wave, supporting H1. This pathway may reflect a coping-based transition from school distress to digital self-regulation. School refusal often occurs when school contexts elicit intense negative affect. These emotions may include anxiety about evaluation, fear of failure, shame, or interpersonal threat (Gonzálvez et al., 2020). Avoiding school can immediately reduce exposure to these aversive cues. This immediate relief creates a strong learning signal, which reinforces avoidance tendencies (Gonzálvez et al., 2018). Such short-term avoidance may also create conditions that increase vulnerability to subsequent PSMU within the same adolescent. It reduces structure, reduces adult monitoring, and increases discretionary time. It can also reduce access to offline support, such as teachers and classmates. Within that “unstructured and distressed” window, social media may become a convenient regulation tool (Odgers and Jensen, 2020). Social media is portable, private, and always available. It offers rapid distraction without requiring planning or skills. Its content stream is unpredictable, which sustains attention through intermittent reward. Notifications, short videos, and social feedback deliver fast emotional shifts. These features can down-regulate distress in the moment, even when the stressor remains unresolved (Kross et al., 2013). For adolescents, this can feel like relief, not avoidance, which increases repeated use.

A key mechanism may involve negative reinforcement, although this interpretation remains speculative. Adolescents do not need to solve school problems to feel better. They only need to disengage from school cues and re-engage with online cues (Fujita et al., 2022). Over time, the adolescent may learn a repeated sequence: school threat → avoidance → scrolling → relief. Each step becomes easier, because the platform lowers the effort required for mood change. This process can transform situational coping into habitual reliance (Shannon et al., 2022). That transition aligns with how PSMU develops as a pattern of dysregulated use (Lu et al., 2025). At the within-person level, temporary increases in school refusal may also contribute to later PSMU through social processes. When adolescents miss school, they may worry about peer evaluation and missing social information. Social media can reduce that uncertainty by offering constant updates. It can also offer safe connection without face-to-face pressure (Przybylski et al., 2013). This is important for adolescents who feel judged or excluded at school. Online interaction reduces immediate interpersonal risk and increases perceived control (Tidwell and Walther, 2002). However, the same reliance can heighten sensitivity to online approval. That sensitivity can strengthen compulsive checking and preoccupation.

The reverse within-person association, i.e., from PSMU to later school refusal, was also significant, supporting H2. It may reflect functioning changes that are associated with greater difficulty attending school. PSMU has been linked to sleep disruption through late-night engagement and notification-driven awakenings (Fobian et al., 2016). Poor sleep increases irritability, anxiety, and stress reactivity the next day. It also reduces executive control and attention, which are needed for school demands (Khan et al., 2024). The adolescent may then experience classes as more overwhelming and less manageable. That experience can increase anticipatory anxiety and avoidance the next morning. Over repeated cycles, school refusal may become more likely following increases in PSMU. PSMU may also affect school refusal by reshaping motivation and reward expectations. Many school tasks offer delayed rewards and require sustained effort. Social media offers immediate reward, rapid novelty, and low entry cost (Wadsley et al., 2022). Frequent exposure to that reward pattern may contribute to perceiving school as comparatively dull. The adolescent may perceive school tasks as unusually effortful, even when tasks are typical. This can lower tolerance for frustration and reduce persistence (Zhang and Jiang, 2023). When effort feels intolerable, avoidance becomes a more attractive option. School refusal may then serve as a way to escape effort, not only distress.

Another plausible mechanism is social comparison and self-evaluation. Social media intensifies upward comparison through curated peer content. This can increase shame, body dissatisfaction, or fear of judgment for some adolescents (Bonfanti et al., 2025). Those feelings can carry into school contexts where peers are physically present. The adolescent may then anticipate evaluation and rejection at school. That anticipation can heighten avoidance motivation and emotional distress (Blakemore and Mills, 2014). In this way, PSMU may be associated with heightened school refusal through heightened social threat perception. This pathway is consistent with adolescents’ sensitivity to peer status.

Importantly, these associations were observed at the within-person level, indicating dynamic processes beyond stable individual risk. Although the standardized cross-lagged coefficients may appear modest, recent methodological work by Orth et al. (2024) suggests that cross-lagged effects of approximately 0.03, 0.07, and 0.12 can be interpreted as small, medium, and large, respectively, in longitudinal panel models. Within this framework, the effects from school refusal to later PSMU (*β* = 0.09–0.10) fall within the medium range, whereas the effects from PSMU to later school refusal (*β* = 0.14) reach or slightly exceed the large range. Given that RI-CLPM provides conservative within-person estimates, these coefficients indicate that the observed associations are practically meaningful.

### 4.2 Significant sex differences

Multi-group analyses indicated significant sex differences in the reciprocal within-person associations, supporting H3. Importantly, both cross-lagged directions were significant for males and females, but their magnitudes diverged. School refusal predicted subsequent PSMU for both males and females, with consistently larger coefficients among females. This pattern suggests that within-person increases in school-related avoidance may be more likely to translate into subsequent increases in problematic social media engagement among females. One interpretation involves emotion-focused coping and interpersonal sensitivity during school distress (McAlister et al., 2024). When school becomes threatening, females may experience stronger internalizing responses, including worry and rumination. Social media can then serve as an accessible tool for mood repair and reassurance seeking (Fassi et al., 2025). It also allows connection without the immediate pressures of face-to-face evaluation. Over time, this coping function may strengthen a stress–use–relief loop, increasing PSMU risk following spikes in school refusal.

The reverse direction showed the opposite sex pattern. PSMU predicted later school refusal for both sexes, but the effects were stronger among males, and the Wald test supported this difference. For males, PSMU robustly predicted subsequent school refusal, whereas the corresponding effects were smaller but significant for females. This finding implies that increases in PSMU may be more strongly associated with subsequent school refusal at the within-person level among males. A routine-disruption mechanism may help explain this pattern. Periods of elevated PSMU may more strongly displace sleep, homework, and morning preparation among males. Such disruptions can increase fatigue, irritability, and academic difficulties, making school attendance more aversive (Lemola et al., 2015). In addition, reward-oriented engagement with digital content may heighten impatience with slower school rewards. School demands may then feel more effortful and less motivating, increasing avoidance tendencies.

### 4.3 Implications and limitations

The present findings have several important implications. Theoretically, the results extend prior evidence of school refusal and PSMU by demonstrating a reciprocal, within-person process linking the two behaviors over time. By applying RI-CLPM, the study highlights that short-term fluctuations, rather than only stable individual differences, play a meaningful role in adolescent maladjustment. This contributes to a deeper understanding of how school disengagement and digital behaviors interact during adolescence. Practically, the findings suggest that school refusal and PSMU should be addressed jointly in prevention and intervention efforts. Changes in either domain may signal emerging risk in the other, even when overall levels appear moderate. The identified sex-specific patterns further highlight the need for tailored approaches. For females, interventions focusing on school-related stress, emotional coping, and peer support may help reduce reliance on social media for regulation (Hou et al., 2019). For males, strategies targeting problematic social media habits, sleep routines, and daily structure may be especially effective in preventing subsequent school avoidance (Kosola et al., 2024).

Several limitations should be considered when interpreting the findings. First, all variables were assessed using self-report measures, which may be influenced by recall bias or social desirability. Future studies should incorporate multi-informant reports or objective indicators, such as school attendance records or digital use logs. Second, although the three-wave longitudinal design strengthens temporal inference, the six-month intervals may not capture shorter-term dynamics between school refusal and PSMU. The choice of a 6-month interval was intended to capture relatively stable developmental changes across the school year, rather than momentary fluctuations. However, more intensive designs, such as diary or ecological momentary assessment methods, could provide finer-grained insights into daily processes and help determine whether the observed within-person associations operate over shorter time scales. Third, the sample was drawn from four middle schools in a single region of China (Zhejiang Province) using convenience sampling, which may limit the generalizability of the findings to other cultural or educational contexts. Specifically, the results may not generalize to adolescents in other provinces, different grade levels (e.g., primary or high school students), other types of schools (e.g., vocational schools), or different urban–rural contexts. Fourth, although RI-CLPM reduces confounding by stable traits, it does not fully account for unmeasured time-varying confounders that may influence school refusal and PSMU. For example, fluctuations in depressive symptoms, bullying victimization, family stress, or other acute contextual stressors may co-occur with changes in both constructs over time, thereby potentially biasing the estimated within-person associations. Future research should incorporate such time-varying covariates.

## 5 Conclusion

This study provides evidence that school refusal and PSMU are dynamically associated at the within-person level during adolescence. Temporary increases in either behavior predict subsequent increases in the other, suggesting reciprocal within-person processes over time. Importantly, the strength of these reciprocal pathways differs by sex. School refusal more strongly predicts later PSMU among females, whereas PSMU more strongly predicts later school refusal among males. These findings highlight the importance of considering both behavioral domains together and recognizing sex-specific developmental pathways. By identifying distinct leverage points for intervention, the study contributes to a deeper understanding of adolescent school disengagement and problematic digital behavior.

## Data Availability

The raw data supporting the conclusions of this article will be made available by the authors, without undue reservation.
